# Bacteria of the *Burkholderia cepacia *complex are cyanogenic under biofilm and colonial growth conditions

**DOI:** 10.1186/1471-2180-8-108

**Published:** 2008-06-27

**Authors:** Ben Ryall, Xiaoyun Lee, James EA Zlosnik, Saiko Hoshino, Huw D Williams

**Affiliations:** 1 Department of Life Sciences, Division of Biology, Faculty of Natural Sciences, Imperial College London, Sir Alexander Fleming Building, London, SW7 2AZ, UK

## Abstract

**Background:**

The *Burkholderia cepacia *complex (Bcc) is a collection of nine genotypically distinct but phenotypically similar species. They show wide ecological diversity and include species that are used for promoting plant growth and bio-control as well species that are opportunistic pathogens of vulnerable patients. Over recent years the Bcc have emerged as problematic pathogens of the CF lung. *Pseudomonas aeruginosa *is another important CF pathogen. It is able to synthesise hydrogen cyanide (HCN), a potent inhibitor of cellular respiration. We have recently shown that HCN production by *P. aeruginosa *may have a role in CF pathogenesis. This paper describes an investigation of the ability of bacteria of the Bcc to make HCN.

**Results:**

The genome of *Burkholderia cenocepacia *has 3 putative HCN synthase encoding (*hcnABC*) gene clusters. *B. cenocepacia *and all 9 species of the Bcc complex tested were able to make cyanide at comparable levels to *P. aeruginosa*, but only when grown surface attached as colonies or during biofilm growth on glass beads. In contrast to *P. aeruginosa *and other cyanogenic bacteria, cyanide was not detected during planktonic growth of Bcc strains.

**Conclusion:**

All species in the Bcc are cyanogenic when grown as surface attached colonies or as biofilms.

## Background

The genus *Burkholderia *is a group of Gram-negative, non-spore forming β-proteobacteria with huge metabolic potential and wide ecological diversity [1]. The *Burkholderia *genus comprises more than 40 species, which range from plant symbionts to commercially important rice pathogens and from opportunistic pathogens of humans to the deadly and potential bio-warfare agent *B. pseudomallei *[1,2].

The *Burkholderia cepacia *complex (Bcc) is a collection of nine genotypically distinct but phenotypically similar species within the *Burkholderia *genus [3,4]. The origins of the Bcc stem from a seminal study by Vandamme *et al*. [5] when 128 *B. cepacia *strains were analysed by genetic and phenotypic means and found to fall into at least five genomic species or genomovars. Over the coming years four other genomovars were assigned to the Bcc complex and each genomovar has now been assigned with a distinct species name, which has replaced the use of the genomovar nomenclature [3,4].

The Bcc show substantial metabolic diversity, and have a relatively unusual (for bacteria) multireplicon genomic arrangement, with all species having more than one chromosome and large genomes ranging from 6 to 9 Mb [4]. The Bcc show wide ecological diversity and include species that are used for promoting plant growth and bio-control as well species that are opportunistic pathogens of vulnerable patients [1,4].

Over recent years the Bcc have emerged as problematic pathogens of the CF lung. All of the species in the Bcc have been isolated from CF lung infections, but the most prominent is *B. cenocepacia*, which accounts for around 70% of all Bcc infections of the CF lung [4,6,7]. CF patients usually acquire Bcc infections later in the course of the disease, often once they are already chronically colonised by *P. aeruginosa *[7]. Similar to *P. aeruginosa*, Bcc strains can form chronic, persistent CF lung infections which are intractable to antibiotic therapy [6,7]. However, in contrast to *P. aeruginosa*, infection can vary from the fairly benign to a rapid respiratory failure and death, referred to as "cepacia syndrome" [8,9]. The diverse pathogenicity in CF appears to be strain rather than species specific with patient factors probably also playing a part since variability is seen in clinical outcome between patients infected with the same strain [4]. Bcc strains possess a number of potential virulence factors that may play a role in CF infection including; LPS, extracellular protease and the ability to form biofilms [4,10]. Similar to *P. aeruginosa*, most of these factors are under the control of quorum sensing, with all species in the Bcc possessing an N-acyl-homoserine lactone signal molecule based system known as the CepI/CepR quorum sensing system [11,12].

*B. cenocepacia *and other members of the Bcc are able to co-colonise the CF lung with *P. aeruginosa *[6,7]. We have recently shown that *P. aeruginosa *infection of the CF lung is associated with the accumulation of cyanide at a mean concentration of 72 μM in the sputum, which correlates with a decline in lung function in CF patients [13]. Under these conditions of cyanide accumulation in the sputum Bcc can co-colonise with *P. aeruginosa*. Therefore, *B. cenocepacia *may also be resistant to the toxic effects of cyanide. Indeed high level cyanide tolerance has been reported in a *B. cepacia *strain [14]. One reason why an organism may be tolerant to cyanide is that it is itself capable of producing cyanide.

The aim of this work was to determine whether Bcc complex bacteria are able to make cyanide.

## Results

### *B. cenocepacia *has three potential homologues of the *P. aeruginosa *cyanide synthase

**Figure 1:**
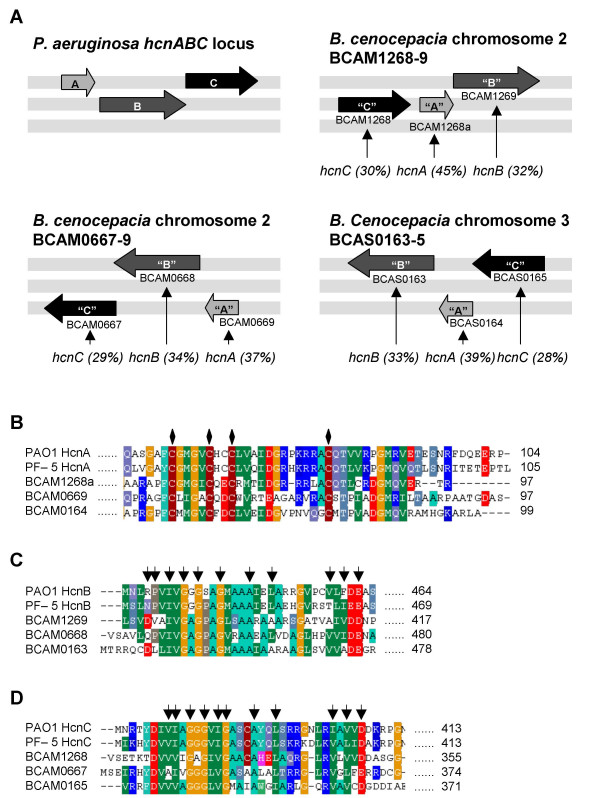
**Putative *hcnABC *genes in *B. cenocepacia***. **A**. The *P. aeruginosa *cyanide synthase enzyme complex is coded for by the *hcnABC *gene locus. *B. cenocepacia *has three sets of possible homologues of the *P. aeruginosa hcnABC *genes. The organisation of the *P. aeruginosa hcnABC *locus is shown in the top left panel followed by the three sets of *B. cenocepacia *putative homologues; two of which are on chromosome two and one on chromosome three. The grey bars indicate the reading frame with the top being +/- 1, middle +/- 2 and bottom +/- 3; the arrows indicate if the genes are on the coding (left to right) or complement (right to left) strands. The percentage identity of the product of each putative gene to their *P. aeruginosa *HcnABChomologue is shown in labels underneath each panel. The Putative Bcc cyanide synthase proteins have conserved Fe-S (HcnA) and ADP binding motifs (HcnB and HcnC). **B**., **C**. and **D**. ClustalX alignments of putative *B. cenocepacia *J2315 cyanide synthase protein sequences with hydrogen cyanide synthases from *P. aeruginosa *(PAO1) and *P. fluorescens *(PF-5). **B**. Sequence of HcnA C-terminal from PAO1 and PF-5 aligned with *B. cenocepacia *putative proteins, BCAM01268a, BCAM0669 and BCAS0164. Diamonds indicate a potential Fe-S binding site at 4 cysteine residues in HcnA. **C**. Sequence of the start of HcnB from PAO1 and PF-5 aligned with *B. cenocepacia *putative proteins BCAM01269, BCAM0668 and BCAS0163. **D**. Sequence from the start of HcnC sequence from PAO1 and PF-5 aligned with *B. cenocepacia *putative proteins BCAM01268, BCAM0667 and BCAS0165. Arrows indicate eleven amino acid ADP-binding motif in HcnB and HcnC.

Cyanide production in *P. aeruginosa *requires the hydrogen cyanide synthase enzyme complex encoded by the *hcnABC *gene locus. We searched the genome sequence of *B. cenocepacia *J2315 [15] to see if the bacterium possessed genes that could potentially code for a cyanide synthase. A TBLASTN search of the *B. cenocepacia *genome using the sequence of the *P. aeruginosa *HcnA, HcnB and HcnC proteins [16] revealed that there were three possible homologues of the *P. aeruginosa *cyanide synthase in *B. cenocepacia*, two on chromosome 2 and one on chromosome 3, the organisation of which are shown in Fig. 1A. Several motifs are believed to be important to hydrogen cyanide synthase function [17]. In HcnA there is a motif containing four cysteine residues believed to be a binding site for an Fe-S cluster, in HcnB and HcnC there is an eleven amino acid motif defined as an ADP-binding motif [17]. These motifs are conserved in each of the three putative HCN synthase clusters in J2315. (Figure 1B, C and 1D)

### Cyanide is not detected in liquid cultures of *B. cenocepacia *strains

We looked for cyanide production by *B. cenocepacia *K56-2 and J2315 growing in LB medium, sampling throughout exponential growth and up to 35 h into stationary phase. Cyanide was not detected in any samples during growth at 30°C or 37°C. The pH of the culture media was measured and was always around pH 8, which is the same as that seen for *P. aeruginosa *cultures in which cyanide is readily detected [18]. This suggests that cyanide was not being lost from the culture as hydrogen cyanide gas due to acidification of the culture medium. Glycine is the precursor for hydrogen cyanide synthesis in *P. aeruginosa *and addition of glycine to medium stimulates *P. aeruginosa *cyanide production; methionine also enhances cyanide production [19,20]. However, no cyanide was detected when *B. cenocepacia *K56-2 and J2315 were grown in LB broth + glycine (12.5 mM) and/or methionine (5 mM). Oxygen is a regulator of cyanide synthesis in *P. aeruginosa*, with synthesis being promoted at low oxygen levels, and so we looked at whether varying oxygen availability led to cyanide formation in liquid culture [17]. However, despite varying the O_2_-transfer coefficient (k_L_a) values from 87.4 h^-1 ^(high) to 11.5 h^-1 ^(low), no cyanide was detected at any stage of growth [21] (data not shown). We conclude that planktonic cultures of *B. cenocepacia *strains K56-2 and J2315 did not accumulate cyanide at detectable levels, which is in contrast to *P. aeruginosa *which accumulates cyanide to a concentration of 300–500 μM under identical growth conditions [19,22,23].

### Cyanide is produced by *B. cenocepacia *strains J2315 and K56-2 when grown on solid medium

**Figure 2:**
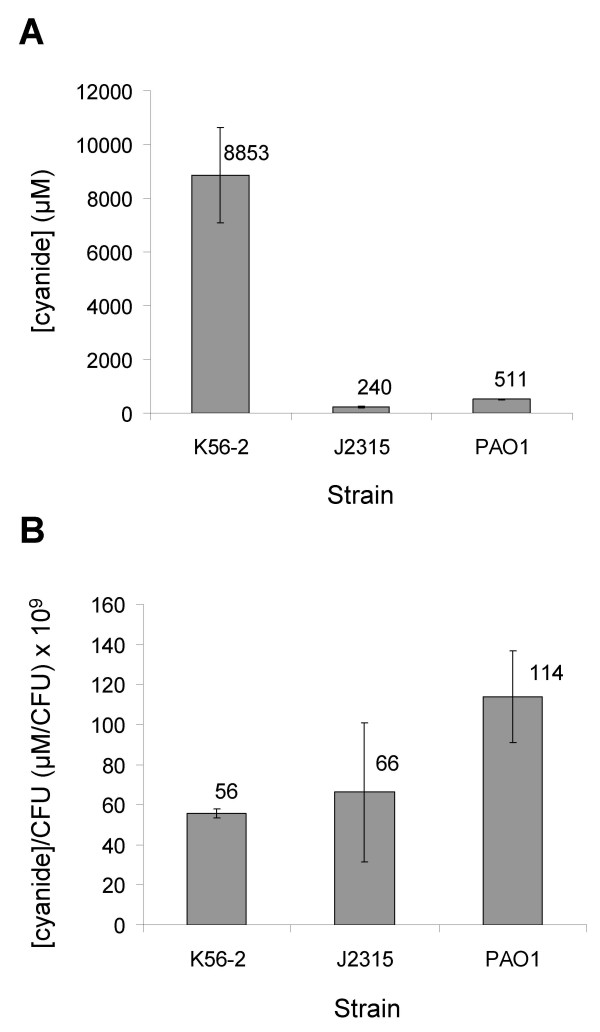
**Cyanide production by *P. aeruginosa *and *B. cenocepacia grown *on solid media**. **A**. Concentration of cyanide given off by plate grown culture and trapped in NaOH. **B**. Concentration of trapped cyanide normalised to the CFU count for the plate. Values are the averages of 3 independent replicates with SE error bars.

Cyanide production by *P. aeruginosa *can also be assayed from cultures grown on solid media [24-26]. Therefore, *B. cenocepacia *was grown on LB agar medium and any cyanide given off during colonial growth trapped and assayed. Under these colonial growth conditions cyanide production was detected from both *B. cenocepacia *K56-2 and J2315. The concentrations of cyanide trapped were 8.9 mM from K56-2 and 240 μM from J2315, while from *P. aeruginosa *PAO1 it was 511 μM (Fig. 2A). However, when normalised to the CFU count of the culture cyanide production from both *B. cenocepacia *strains was similar to that of *P. aeruginosa *(Fig. 2B). This demonstrated that *B. cenocepacia *strains were cyanogenic and that the levels of cyanide produced were comparable to those produced by *P. aeruginosa*.

### Cyanide is produced by all species of the Bcc when grown on solid media

**Figure 3:**
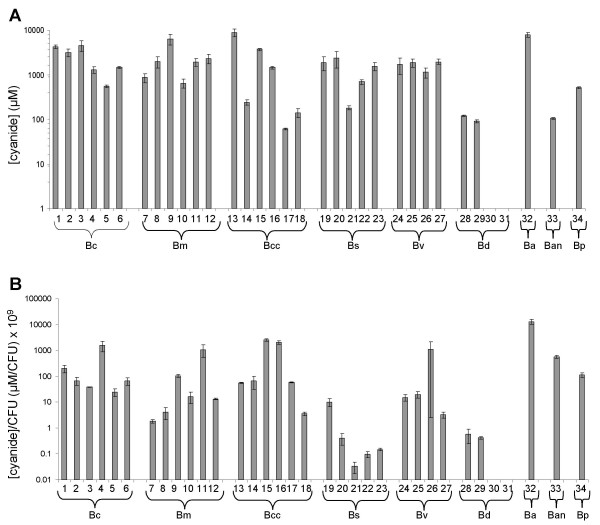
**Cyanide production by members of the Bcc grown on solid media**. Concentration of cyanide given off by plate grown cultures of Bcc strains. **A**. Concentration of cyanide trapped in NaOH. **B**. Cyanide concentrations from A normalised to the CFU count for the plate. Values are the averages of 3 independent replicates with SE error bars. B.c., *B. cepacia*; B.m., *B. multivorans*; B.cc., *B. cenocepacia*;B.s., *B. stabilis*, B.v., *B. vietnamensis*; B.d., *B. dolosa*; B.a., *B. ambifaria*; B.an, *B. anthina*; B.p., *B. pyrrocinia*. The numbers under the axis correspond to stain names (for key to strain names see Table 1).

We next screened other members of the Bcc for cyanide production. A total of 34 strains with at least one representative from each species (genomovar) were assayed using the LB agar plate method (Table 1). Every strain tested was cyanogenic except for two *B. dolosa *strains, LMG18943 and LMG18944 (numbers 30 and 31 in Fig. 3). There was a greater than 2 log variation (~60 μM to 19 mM) in the concentrations of cyanide trapped from the different Bcc species (Fig. 3A), and a greater than 5 log variation (0.03 μM/10^9 ^CFU to 13000 μM/10^9 ^CFU) when the concentrations were normalised to the CFU counts obtained from the plates (Fig. 3B). In this assay a *P. aeruginosa *Δ*hcn *strain produced no detectable cyanide [23]. The limited number of replicates for some strains meant that mean cyanide production could only be compared between *B. cepacia*, *B. multivorans*, *B. cenocepacia *and *B. stabilis *(nonparametric ANOVA, Kruskal – Wallis test). The mean normalised cyanide concentration for the *B. stabilis *strains (2.2 μM/10^9 ^CFU) was significantly lower than the means for both the *B. cepacia *strains (325 μM/10^9 ^CFU) and *B. cenocepacia *strains (792 μM/10^9 ^CFU) (Dunn's multiple comparisons test, p < 0.05 for both), no other significant differences were seen in mean cyanide production between strains. All strains were also assayed for cyanide production in liquid culture, but like K56-2 and J2315 none of them tested positive.

**Table 1:** Bacterial strains used in this study.

Number in figure 3	Strain	Description
	***P. aeruginosa***	
	PAO1	Wild-type, prototrophic strain
	***Burkholderia cepacia***	
1	LMG6993	Environmental strain, soil
2	LMG6988	Clinical isolate, leg wound
3	LMG6963	Environmental isolate, soil
4	LMG1222	Environmental isolate, onion
5	LMG18821	Clinical isolate, CF lung
6	LMG14095	Clinical isolate, CF lung
	***Burkholderia multivorans***	
7	LMG13010	Clinical isolate, CF lung
8	LMG16660	Clinical isolate, CF lung
9	LMG16665	Clinical isolate, brain abscess
10	LMG17588	Environmental isolate, soil
11	LMG18822	Clinical isolate, CF lung
12	LMG18945	Clinical isolate, CF lung
	***Burkholderia cenocepacia***	
13	K56-2	Clinical isolate, CF lung
14	J2315	Clinical isolate, CF lung
15	LMG16654	Clinical isolate, CF lung
16	LMG16659	Clinical isolate, CF lung
17	LMG18830	Clinical isolate, CF lung
18	LMG18827	Clinical isolate, CF lung
	***Burkholderia stabilis***	
19	LMG6997	Clinical isolate, ear
20	LMG7000	Clinical isolate, blood
21	LMG14291	Clinical isolate, CF lung
22	LMG14294	Clinical isolate, CF lung
23	LMG18138	Clinical isolate, CF lung
	***Burkholderia vietnamensis***	
24	LMG18835	Clinical isolate, CF lung
25	LMG6998	Clinical isolate, blood
26	LMG10928	Environmental isolate, rice
27	LMG16232	Clinical isolate, CF lung
	***Burkholderia dolosa***	
28	LMG18941	Clinical isolate, CF lung
29	LMG18942	Clinical isolate, CF lung
30	LMG18943	Clinical isolate, CF lung
31	LMG18944	Clinical isolate, CF lung
	***Burkholderia ambifaria***	
32	LMG19182	Environmental isolate, pea rhizosphere
	***Burkholderia anthina***	
33	LMG20983	Clinical isolate, CF lung
	***Burkholderia pyrrocinia***	
34	LMG14191	Environmental isolate, soil

All Bcc strains were obtained from the BCCM/LMG collection, University of Ghent, Ghent, Belgium.

In summary, all nine species in the Bcc are capable of producing cyanide when grown on solid media. The level of cyanide production in the Bcc appears to be strain rather than species specific.

### Is cyanide production in *B. cenocepacia *a biofilm specific phenotype?

**Figure 4:**
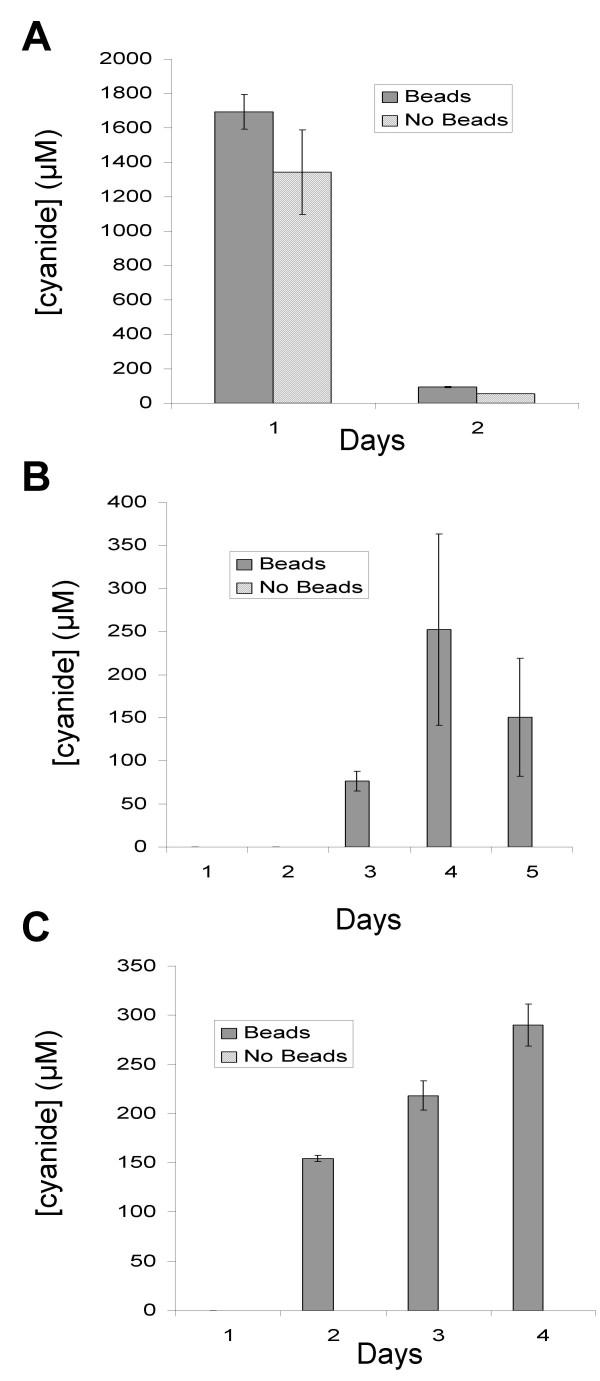
**Cyanide production from glass bead "biofilm" model**. Concentration of cyanide given off by glass bead biofilm cultures over 6 hours and trapped in 1 ml 4 M NaOH. **A**., **B**. and **C**. cyanide concentration trapped in NaOH for *P. aeruginosa *PAO1, *B. cenocepacia *J2315 and *B. cenocepacia *K56-2 respectively. Solid bars: with glass beads, diagonal striped bars: without glass beads. Measurements are averages of 3 independent replicates with SE error bars.

The finding that cyanide was not detected in liquid cultures of *B. cenocepacia *and other members of the Bcc complex, but was readily detected from plate grown cultures suggested that cyanide production in Bcc is a surface attached phenotype. Bcc can grow as a biofilm [10,27] and we were interested to test whether *B. cenocepacia *produces cyanide when growing surface-attached as a biofilm. We grew Bcc strains as biofilms on 6 mm diameter glass beads immersed in LB broth in a Petri dish, which provided a large surface area for growth. Biofilm growth was detected visually and by crystal violet staining [10,27]. Cyanide production was assessed by trapping cyanide given off from the biofilm culture in 4 M NaOH. Control cultures comprised Petri dishes containing bacterial culture without glass beads. Figure 4A shows the data obtained for the *P. aeruginosa *PAO1. Cyanide was detected in the presence and absence of glass beads, consistent with *P. aeruginosa *being able to make HCN during planktonic growth, but after three days incubation cyanide was no longer detected (data not shown).

For the *B. cenocepacia *strains J2315 and K56-2 cyanide was only detected from the glass bead biofilm cultures and not from the control planktonic cultures (Fig. 4B and 4C). Cyanide was not detected until day 2 or day 3 for K56-2 and J2315 respectively. This is consistent with cyanide production resulting from biofilm growth, which takes time to establish on the glass bead surface.

Providing *B. cenocepacia *strains with a large surface area suitable for cell attachment/biofilm formation led to the detection of cyanide from the culture. This, together with the findings that Bcc strains produce detectable cyanide when grown on solid media but not in liquid culture, suggests that cyanide production in *B. cenocepacia *and other members of the Bcc is enhanced in surface attached cells and is a biofilm specific phenotype.

## Discussion

In this paper we have demonstrated that *B. cenocepacia *and each of the eight other species of the *Burkholderia cepacia *complex can produce cyanide. This is the first time that any member of the Bcc has been reported to produce cyanide, although there are published reports of unsuccessful attempts to detect cyanide production by *B. cepacia *strains [28,29]. We can only surmise that strain or growth condition differences prevented the detection of cyanide production in these previous studies. Like *P. aeruginosa*, members of the Bcc are important pathogens of the CF lung [7,30]. Therefore, this finding is potentially significant in terms of CF lung infection, especially in light of our recent finding that cyanide accumulates in the lungs of *P. aeruginosa*-infected CF patients and is associated with a decline in lung function [13]. An interesting possibility is that Bcc infecting the CF lung makes cyanide, and that cyanide production provides a survival and/or colonisation advantage to CF-lung infecting bacteria.

Cyanide is a potent inhibitor of cellular respiration; it binds to and inhibits ferric iron containing enzymes including cytochrome *c *oxidase. Bacterial cyanide production during lung infection would potentially damage local host tissue and possibly inhibit immune cell function. Thus, it is intriguing that the two major infecting bacteria of the CF lung are able to produce cyanide.

The variation in cyanide levels was strain rather than species specific, as is the pathogenicity of members of the Bcc in CF lung infection [4] and in the pathogenicity to *C. elegans *[31].

Cyanide production during planktonic growth in liquid culture was not detected for any members of the Bcc. It is possible that cyanide was produced but that it did not accumulate to a high enough level to be detected. However, in the plate cyanide assay the concentration of cyanide measured for many strains was significantly higher than that seen for *P. aeruginosa *PAO1, which does accumulate cyanide at readily detectable levels in liquid culture [18,19,23]. This suggests that Bcc strains are capable of producing cyanide at levels comparable to *P. aeruginosa *and that the absence of detectable cyanide in liquid cultures of the Bcc was not just because the strains are low level cyanide producers. The data presented in this paper is consistent with cyanide production in the Bcc being induced during colonial growth. The most common type of biofilm and the form usually investigated are those occurring between a solid/liquid interface, but the description can apply equally to the solid/air interface of colonial growth on an agar plate. It follows that cyanide production in the Bcc may be induced during biofilm growth. To test whether this occurs in *B. cenocepacia*, a solid/liquid interface biofilm model was used, which employed 6 mm glass beads in a Petri dish. *B. cenocepacia *produced cyanide in this model, and it took at least two days before cyanide was detected, suggesting that full establishment of the biofilm was necessary for cyanide production. These data support the view that cyanide production in *B. cenocepacia *is a biofilm specific phenotype. Work is now needed to determine the molecular mechanisms underlying cyanide production in the Bcc.

It is possible that cyanide production in biofilms is used to inhibit the growth of competing bacteria. Naturally occurring biofilms are usually made up of mixed populations of bacterial species; therefore it is likely that anti-microbial products will play a role in interspecies competition in biofilms. Indeed pyocyanin production by *P. aeruginosa *was found to inhibit *B. cepacia's *ability to form mixed biofilms with *P. aeruginosa *[27].

## Conclusion

This paper reports that *B. cenocepacia *and the other members of Bcc produce cyanide. As such, they join a limited number of organisms that are capable of producing this potent inhibitor of aerobic respiration, but uniquely, to date they are the only bacteria described as being cyanogenic only during surface attached growth.

## Methods

### Strains, growth media and culture conditions

The bacterial strains used in this study are listed in Table 1. 1 ml of an overnight starter culture grown in Luria-Bertani broth (10 g/l tryptone (Sigma), 5 g/l yeast extract (Sigma), 5 g/l NaCl), was used to inoculate 50 ml of LB broth in a 250 ml flask, which was then incubated in an orbital shaker at 200 rpm at 37°C. To look at the effects of oxygen on the ability of Bcc to produce cyanide during liquid growth, cultures were grown at three O_2_-transfer coefficient (k_L_a) values: high (87.4 h^-1^), medium (27.8 h^-1^) and low (11.5 h^-1^) [21]. This was achieved by using identical 250 ml flasks, shaking at 200 rpm, but altering the medium volume [21].

### Cyanide measurements

An ELIT (ISM-146 CN) cyanide ion-selective micro-electrode (Lazar Research Laboratories, L.A., CA, USA) was used for measuring cyanide concentration in culture supernatants as described previously [23].

### Agar plate cyanide assay

This method was adapted from [25]. LB agar plates were inoculated by spreading with 100 μl of a 1:10 dilution of mid-log phase culture (OD_600 _= 0.5) and incubated overnight at 37°C. Plates, with lids removed, were then placed inside sterile 140 mm Petri dishes alongside a weigh boat containing 1 ml of 4 M NaOH. The 140 mm Petri dish was then sealed with Parafilm and incubated for 4 hours at 37°C. The concentration of cyanide (given off by the culture) trapped in the NaOH was then measured and normalised to the CFU present on the plate. CFU counts were obtained by scraping the culture off the plate and suspending it in LB, dilutions were then plated out and colonies counted.

### Glass bead biofilm assay

The glass bead biofilm apparatus consisted of a 90 mm Petri dish filled with 48 g of 6 mm glass beads (Fluka), which was sufficient to cover the bottom of the Petri dish in a single layer. It was inoculated with 12 ml of a 1:10 dilution of mid-log phase culture (OD_600 _= 0.5). After over night incubation at 37°C the culture medium was removed and replaced with fresh LB broth. The biofilm plates were then placed inside 140 mm Petri dishes alongside a weigh boat containing 1 ml of 4 M NaOH. The 140 mm Petri dishes were then sealed with Parafilm and incubated for 4 hours at 37°C, and the concentration of cyanide trapped in the NaOH was measured. After cyanide measurement the culture media was removed from the biofilms and replaced with fresh LB broth and the biofilms were incubated at 37°C until the next day, when the process was repeated. Petri dishes without beads were included as controls; these were treated in exactly the same way as the glass bead biofilm plates

## Authors' contributions

BR participated in the design and co-ordination of the study, carried out plate and liquid culture cyanide assays, conceived and developed the glass bead biofilm model, carried out the biofilm cyanide assays, carried out the statistical analysis and drafted the paper. XL carried out plate cyanide assays. SH carried out plate cyanide assays. JZ carried out the bioinformatics/sequence alignments and helped draft the manuscript. HW conceived of the study, participated in its design and co-ordination and wrote the final draft of the manuscript.
